# Adaptive infrared patterns for microscopic surface reconstructions

**DOI:** 10.1007/s11548-024-03242-8

**Published:** 2024-10-09

**Authors:** Srdjan Milosavljevic, Zoltan Bardosi, Yusuf Oezbek, Wolfgang Freysinger

**Affiliations:** Department of Otorhinolaryngology, Medical University of Innsbruck, Innsbruck, Austria

**Keywords:** Adaptive pattern, Stereo reconstruction microscope, Random pattern, Bayesian optimizer, ENT procedures

## Abstract

**Purpose:**

Multi-zoom microscopic surface reconstructions of operating sites, especially in ENT surgeries, would allow multimodal image fusion for determining the amount of resected tissue, for recognizing critical structures, and novel tools for intraoperative quality assurance. State-of-the-art three-dimensional model creation of the surgical scene is challenged by the surgical environment, illumination, and the homogeneous structures of skin, muscle, bones, etc., that lack invariant features for stereo reconstruction.

**Methods:**

An adaptive near-infrared pattern projector illuminates the surgical scene with optimized patterns to yield accurate dense multi-zoom stereoscopic surface reconstructions. The approach does not impact the clinical workflow. The new method is compared to state-of-the-art approaches and is validated by determining its reconstruction errors relative to a high-resolution 3D-reconstruction of CT data.

**Results:**

200 surface reconstructions were generated for 5 zoom levels with 10 reconstructions for each object illumination method (standard operating room light, microscope light, random pattern and adaptive NIR pattern). For the adaptive pattern, the surface reconstruction errors ranged from 0.5 to 0.7 mm, as compared to 1–1.9 mm for the other approaches. The local reconstruction differences are visualized in heat maps.

**Conclusion:**

Adaptive near-infrared (NIR) pattern projection in microscopic surgery allows dense and accurate microscopic surface reconstructions for variable zoom levels of small and homogeneous surfaces. This could potentially aid in microscopic interventions at the lateral skull base and potentially open up new possibilities for combining quantitative intraoperative surface reconstructions with preoperative radiologic imagery.

## Introduction

In many surgical interventions, specifically microscopic ENT surgeries at the lateral skull base, a surgeon would benefit from additional intraoperative data [1] offered by dense and accurate “real-time” surface reconstruction of the operative site. Registration of these with other pre- or intraoperative medical imagery could be beneficial for intraoperative surgeon guidance [2]. In microscopic interventions at the lateral skull base, reconstruction of homogeneous structures like bone or tissue is challenging due varying zoom levels [3], operating room lighting conditions and extreme illumination [4] prone to specularities and shadowing. This significantly affects invariant feature detection, disparity estimation and surface stereo reconstruction [5].

**Fig. 1:**
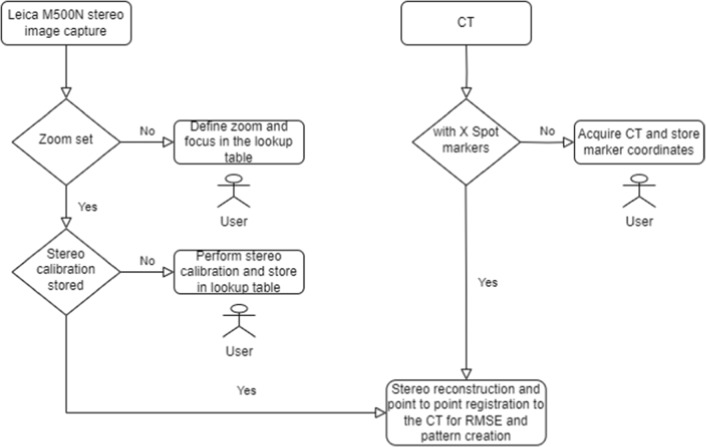
Flow diagram of the system and user interaction for system initialization: multi-zoom calibration, marker detection and MiRe on the left, CT reconstruction and fiducial detection on the right, for co-registration of CT images to MiRe by minimizing RMSE (root mean squared error) via an iterative Bayesian optimizer loop. Required user interactions are shown by the mannequins

Stereoscopic surface reconstruction is a well-established technology that builds on feature matching between stereo image pairs using detectors like SIFT [6] and SURF [7], detecting invariant object features with inhomogeneous structures and matching blocks of pixels locally (block matching, BM or semi-globally (SGBM [8, 9])). BM yields a significantly higher density, but not necessarily a high accuracy [10]; pairing improper features leads to inaccurate reconstructions unsuitable for further quantification of the amount of resected tissue or for further multimodal registration. The FLANN detector [11], optimized with number of trees [12], improves the speed of feature detection, but cannot consider all true positive features due to computational tree limits [13]. Brute force approaches that compare all points to each other are prone to false positives and are computationally expensive [14].

To overcome the challenge of (tissue) homogeneity highly discernible patterns are projected on the objects to be reconstructed. Different methodologies are available for stereo reconstructions and even augmented reality [15–17], paradigmatically in ENT procedures at the lateral skull base. However, these methods are static and are not available for variable zoom and small (anatomical) structures, as is the case in this type of surgeries [18].

Enhancing intraoperative setups with proper illumination requires special methods for feature collection and triangulation. Time of flight [19] requires reliable and static optics (without zoom); structure from motion [20] will need some actions of a surgeon to collect valid data, impacting the clinical workflow and extending the procedure time; trifocal tensor [21] approaches provide a robust but bulky setup, too complex for surgical environments.

Adaptive infrared patterns are promising to enhance stereo matching under realistic operating room conditions to enable dense microscopic reconstructions (MiRe) of homogeneous surfaces [22, 23]. The adaptive pattern reconstruction model, obtained from a Leica surgical stereo microscope, is compared to a model reconstructed from CT images via multimodal registration and point to point comparison. The method is evaluated on a colored realistic 3D print of a human ear for various zoom settings. Different projected patterns and illumination conditions serve assessing reconstruction accuracy aiming at developing additional navigated (ENT) procedures to allow quantitative monitoring surgical procedures of tissue removal during petrous bone surgeries.

## Materials and methods

The application is written for use on Intel i7, 16GB RAM, Nvidia GTX 1060 powered PC using C++ and incorporating VTK [24], ITK [25], OpenCV [26], PCL [27]. It can read DICOM [28] studies, generate 3D models from MiRe and CT, capture microscope images, perform stereo calibration, control zoom, multimodal registration and generate near-infrared (NIR) pattern via Bayesian optimization. Figure 1 represents the workflow.

### Surface model and ground truth CT dataset

**Fig. 2:**
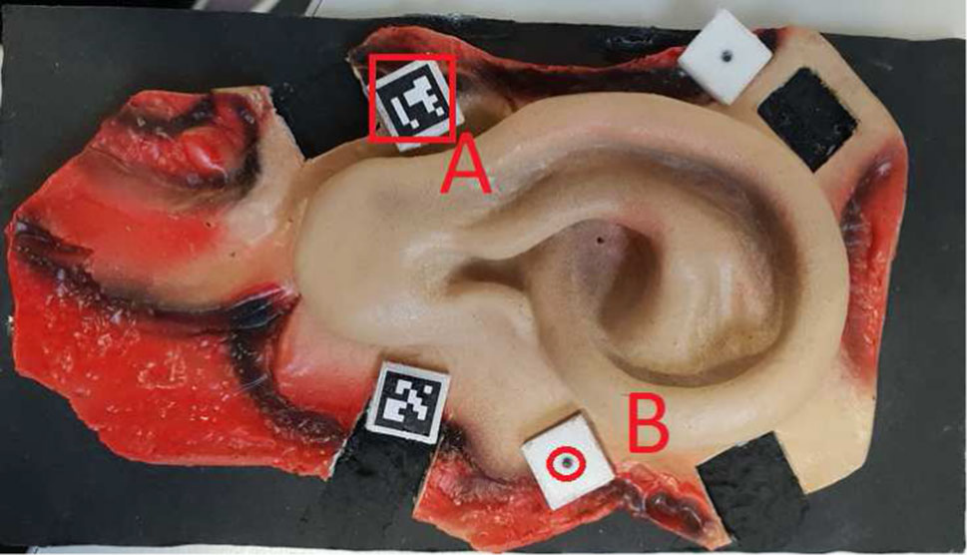
Real scale ear model with ArUco (**A**) and X-spot markers (**B**) in the cube prior to being hidden by the ArUco markers (**B**)

A real-scale realistic and colored plastic model of an adult external human ear ($9 \times 4$ cm) was CT scanned as ground truth. This 3D print is suitable for evaluating surface reconstructions as shape, color, occlusion and illumination are demanding. Four 3D-printed holders different in height and inclination of the uppermost surface are placed on the ear and carry custom-made multimodal markers: an ArUco [29, 30] marker for microscope camera pose estimation and an CT X-Spot spherical markers (1.5 mm diameter, titanium CT markers, Beekley Medical) placed 1 mm below the ArUco marker origin (Fig. 2).

**Fig. 3:**
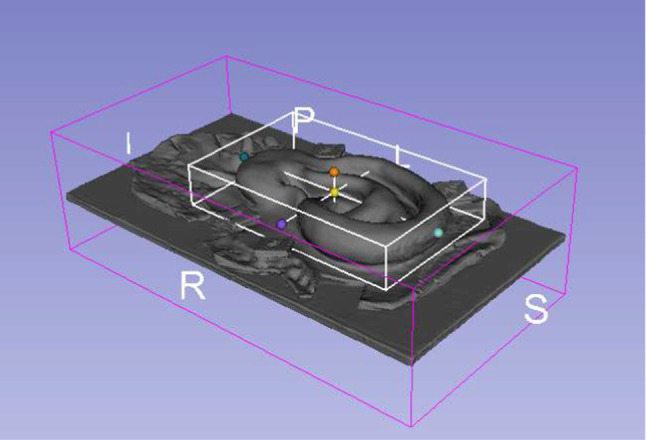
Segmented ground truth ear CT model with the region of interest contained in the white cuboid. The letters indicate patient coordinates. Image created with 3D-Slicer [32]

A ground truth 3D model was created from CT images with 0.6 mm slice thickness windowed at 2800 HU with the Marching cubes algorithm [31]; it is shown in Fig. 3 where the region of interest (ROI) is shown inside the white cuboid. The cuboid volume was used for the MiRe reconstruction error measurements.

### Leica M500N stereo microscope

**Fig. 4:**
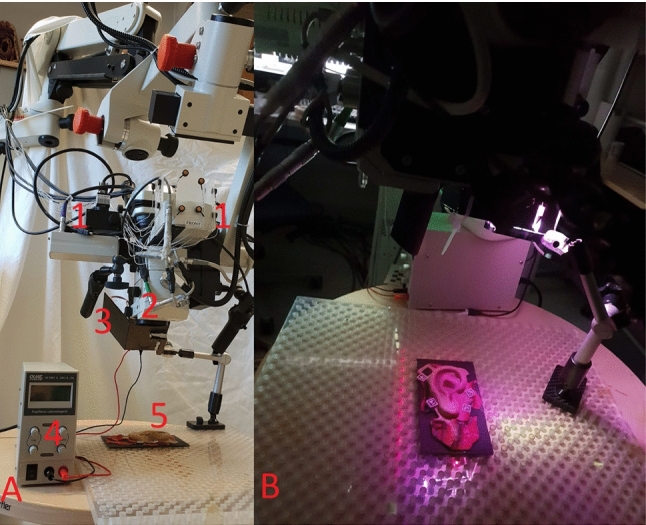
Entire setup. **A** all system components 1: NIR cameras, 2: Microscope lens, 3: NIR projector, 4: NIR power source, 5: Ear model; **B** sample pattern projected on the ear phantom

A Leica M500N surgical stereo operating microscope (Heerbrugg, Switzerland) was equipped with two high-resolution cameras (2456 x 2054 pixels, IDS UI-3080CPM-GL monochrome, IDS GmbH, Obersulm, Germany) connected to the microscope on two beam splitters introduced into the microscope optical path for scene view extraction. These cameras have a maximal NIR sensitivity at 740 nm and provide left and right image pairs of the surgical scene. The NIR adaptive pattern projector is mounted to the body of the microscope to illuminate the microscope’s field of view. Microscope images are transferred via USB 3.0, and zoom and focus are controlled by a CAN-bus interface on the planning station (Fig. 4).

#### Microscope optical calibration

**Fig. 5:**
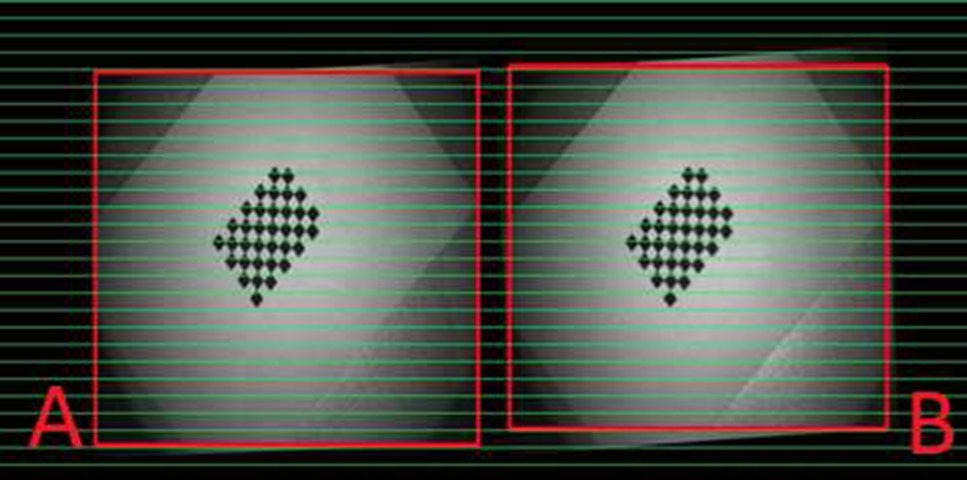
Rectified left and right camera views after successful calibration

The microscope is calibrated at 5 different zoom levels that still allow a focused view on the whole ear model including the markers at the microscope’s working distance (below 20 cm) in both camera views. These boundary conditions restricted the use of zoom levels greater than 1.5. Stereo calibration is done with Zhang’s method [33] by simultaneously taking image pairs (19 pairs per zoom) of a 9 $\times$ 5 checkerboard (3 $\times$ 3 mm squares) for a wide range of translations/rotations (up to $60^\circ$). Intrinsic parameters provide focal length and optical centers of each camera, while extrinsic parameters based on the intrinsic parameters provide relations (rotation and translation) between the two cameras. Both parameters for specific zoom and focus settings are determined and stored in a lookup table. Figure 5 represents an example of rectified pairs; the image fractions used for disparity calculation are marked with red squares.

Disparity was calculated with SGBM [8]. All parameters (i.e., minimum minus disparity, block size=3) were kept constant for all comparison methods mentioned in “Method comparisons” section. Experimental variations of block size did not improve results, but resulted in inferior detection on reflecting homogeneous surfaces and to disappearance of small details in the reconstructions.

### Power source and near-infrared (NIR) projector

NIR light sources for the pattern projector were evaluated with a photo-diode power sensor (Thor labs S121C, USA) at 740 nm and a bandwidth filter ($\ge$700 nm) in an operating room (OR). No other significant light sources were found at this wavelength, with environmental NIR light being $<10 \upmu$W at 740 nm. No significant NIR absorption was measured for the microscope, too. Thus, NIR light at 740 nm was useful for the experiment with the microscope. A temperature controlled LED with custom-made active cooling, powered with a stabilized power supply (QUAT Power LN-3003, Pforring, Germany), overheating protection provided 3W of infrared illumination.

**Fig. 6:**
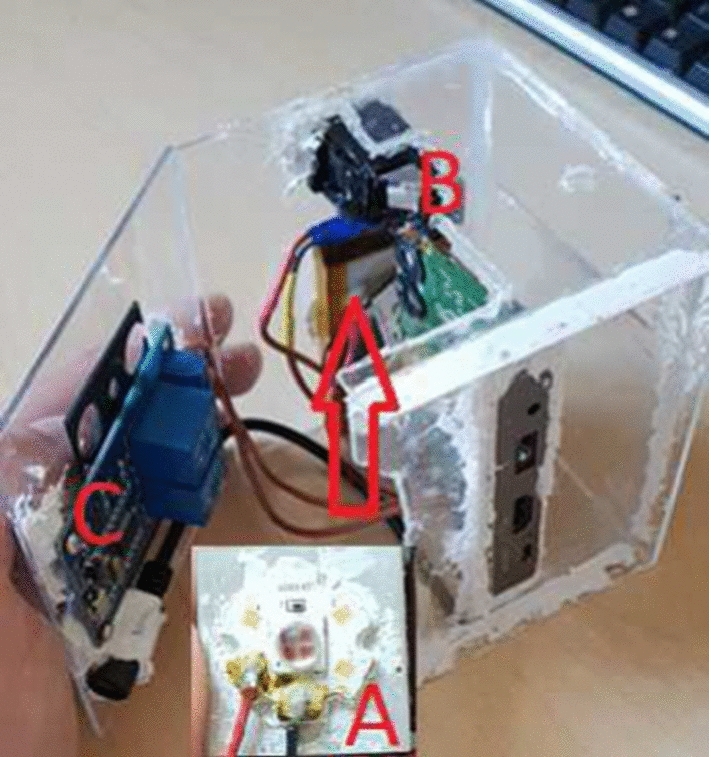
NIR LED with active cooling (**A**), former DLP optics housing to fit the NIR LED (**B**) and relay system (**C**)

The custom-built NIR source illuminates the adaptive pattern projector, a customized APEMAN, DLP (Digital Light Processing), (resolution 800 $\times$ 600 pixels, Apeman International Co., Ltd., Shenzhen, China). All other optical components of the DLP were removed to avoid NIR absorption. NIR LED light intensity output of the projector was measured 800 mW, sufficient for adaptive pattern projection and detection (Fig. 6).

### Rigid body registration and closest point RMSE

ArUco markers are detected in the MiRe reconstruction as points for the rigid body registration to the X Spot markers detected in the CT to set the ground for the further evaluation of the reconstruction quality. The registration is performed with an algorithm from Horn [34] for absolute orientation, implemented to find the transformation from MiRe to CT and prepare two models for the closest point RMSE as an ultimate reconstruction error, explained in “Adaptive pattern generator with Bayesian optimizer” section .

### Adaptive pattern generator with Bayesian optimizer

The adaptive pattern consists of an arrangement of dots in a rectangular area. Dot size (s) and distance (d) are to be optimized for each zoom level, representing significant effort if performed manually with respect to the procedure time, due to different possibilities of dot size and distance to generate the pattern and minimize the reconstruction error. To ease this, a Bayesian optimizer [35] was used. A simple Gaussian process [36, 37] surrogate model with an RBF+Scaling kernel was used to model the mean squared reconstruction error over the reference object. For each iteration, the goal was to find an optimal setting for d and s that minimizes the reconstruction error to our reference CT object providing to the optimizer parameters, x:=$\left[ d, s \right]$ where dot size, s, from 2 to 16 pixels, and distance, d, from 1 to 10 pixels in the pattern. At each evaluation, a MiRe reconstruction was made with the predefined zoom level and NIR projection created with the testing parameters x.

The centers of the ArUco markers were located in the stereo reconstruction (MiRe). With the known relation of the ArUco to the radiolucent marker in the base of the artificial ear, the stereo reconstruction was registered to the 3D reconstructed CT surface, see “Rigid body registration and closest point RMSE” section, for further evaluation and RMSE extraction. Random 5000 points subset was chosen from the reconstructed point cloud ($t_s, s \in \left[ 1.. 5000 \right]$), and for each $t_s$ in the reconstructed point set, the closest point of the CT point set was chosen as a correspondence (marked as $r_s$). The reconstruction error was calculated as the RMSE between $t_s$ and $r_s$, respectively.

$$\begin{aligned} \sum _{s=1}^{5000}\left( \frac{\left| t_s-r_s \right| }{5000} \right) \end{aligned}$$

The Bayesian optimization process was initialized with 10 random evaluations. The acquisition function was chosen to be the expected improvement formulation at each step until the end of the iteration.

The optimized dot distance d and size s values were obtained and stored in a lookup table for all 5 zoom levels investigated before applying changes to the 3D model, as this would impact the MiRe dataset and therefore comparison to the CT.

### Method comparisons

The effects of the different object illumination on the MiRes were investigated in the following experiments. The artificial external ear was placed in the view of the optically calibrated microscope, and the illumination conditions were set using different methods:

- Standard OR environmental light, no microscope light, no extra additional light source.
- Standard microscope illumination.
- Projection of random NIR patterns [18, 38].
- Projection of Bayesian optimized adaptive NIR patterns. [18, 38].

Experiments were performed at 5 zoom levels in which the whole ear model was visible and in focus with 20 cm distance between an ear placed on a table and the microscope objective. The model was manually rotated relative to the microscope to detect possible angulation dependent reconstruction errors due to potential over-, under illumination, occlusions and specularities. Illumination power in the visible and IR domain was constant for all tests. RMSE mean and standard deviation were calculated between CT model and MiRe based on randomly selected, normally distributed 5000 closest points inside the ROI for 5 zoom levels, 4 methods and 10 reconstructions per setting, yielding 200 surface reconstructions as shown in Table 2.

A heat map was generated in the Z-direction to visualize differences in structure recognition and depth estimation of the 3D reconstructions obtained using proposed methods.

For extra validation purposes, the stereo reconstructed 3D surfaces of the ear by the adaptive pattern were compared visually to a 3D surface provided by Carl Zeiss Optotechnik GmbH, Neubeuern, Germany.

## Results

**Fig. 7:**
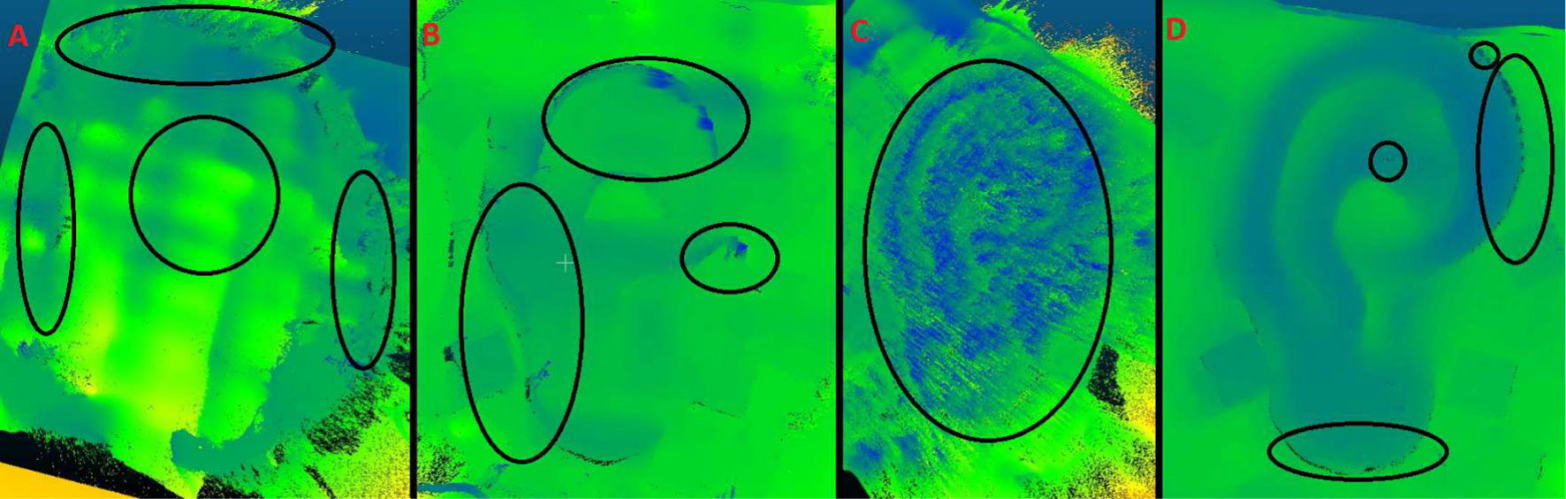
Ear model reconstruction/structures for 4 different methods at zoom level 1.5. Ellipses show areas with lack of correct depth information: **A** microscope light; **B** environmental (OR) light; **C** random/diffractive pattern; and **D** adaptive pattern

Results were collected for the described methods using environmental light showing acceptable overall results as presented in Table 2, having still significant outliers showing bad disparities as in Fig. 7, part B, caused by the light strength and the source position, but also due to the lack of the salient features to compare homogeneous surfaces [39].

Quite on the contrary. We observed a lack of information when using the microscope’s halogen illumination, see Fig. 7, part A. This was not an issue for the other light sources used as their intensity could be down-regulated very quickly and switched to another approach, as proposed in the discussion.

Random NIR pattern was based on a diffractive optics showing promising results as presented in Table 2, but it was impossible to control the distribution or the size of the features, which brought the noise to the reconstruction as shown in Fig. 7, part C.

For the adaptive NIR pattern, Table 1 presents Bayesian optimizer’s worst and best predictions for pattern dot size and separation per zoom level. Table 1 shows better results on higher zoom levels and in Fig. 7, part D. The time to generate MiRe was around 2 s, while CPU time to obtain adaptive pattern parameters, co-registration and calculating the reconstruction error was below 20 s.

First the Bayesian optimizer’s worst and best predictions for pattern dot separation and dot size per zoom level are presented, see Table 1. The time to generate MiRe was around 2 s, while CPU time to obtain adaptive pattern parameters, co-registration and calculating the reconstruction error was below 20 s.

**Table 1:** Bayesian optimizer values per zoom level; the upper and lower lines in the columns show the **best** and the worst results for the adaptive patterns, respectively

Zoom	Dot distance (pixel)	Dot size (pixel)	RMSE (mm)
1.1$\times$	10	1	1.0
	**6**	**2**	**0.7**
1.2$\times$	10	1	1.0
	**14**	**6**	**0.7**
1.3$\times$	9	1	0.9
	**16**	**7**	**0.5**
1.4$\times$	16	1	1.0
	**14**	**5**	**0.5**
1.5$\times$	5	1	0.9
	**15**	**7**	**0.5**

Table 2 represents sum of 10 mean and standard deviation RMSE per zoom level for proposed methods.

**Table 2:** Zoom levels with mean and standard deviation RMSE given in millimeters

Zoom	Mean ± SD (mm)		
	Adaptive	Random	Environmental
1.1	$0.7 \pm 0.4$	$1.9 \pm 1.8$	$1 \pm 0.9$
1.2	$0.7 \pm 0.4$	$1.7 \pm 1.7$	$1.2 \pm 1$
1.3	$0.5 \pm 0.3$	$1.8 \pm 1.8$	$1.1 \pm 1.0$
1.4	$0.5 \pm 0.3$	$1.5 \pm 1.6$	$1.8 \pm 1.5$
1.5	$0.5 \pm 0.3$	$1.6 \pm 1.7$	$1.7 \pm 1.4$

**Fig. 8:**
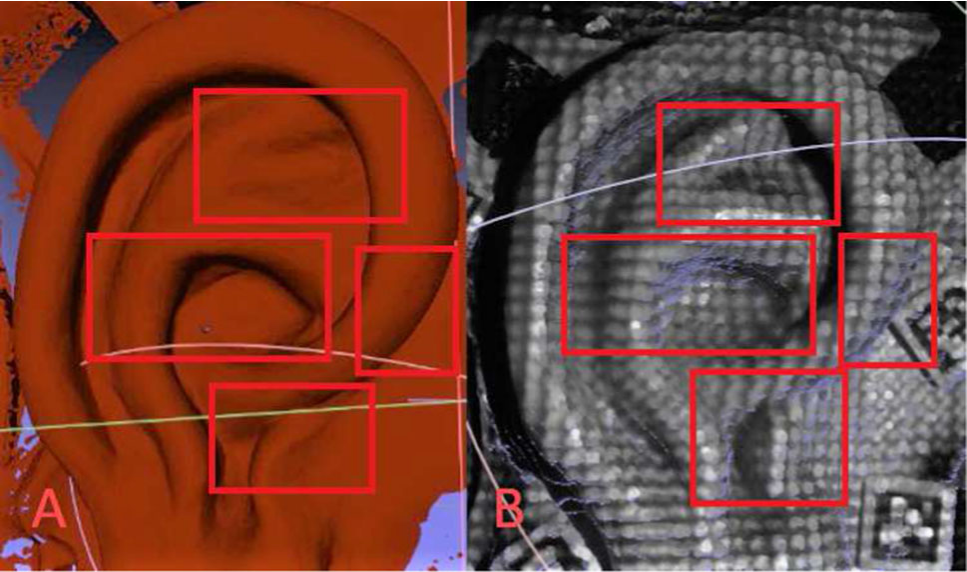
Visual comparison of surfaces from **A** Carl Zeiss Optotechnik 3D surface and **B** adaptive pattern MiRe showing some advantages especially in the regions of concha and incisura

Figure 7 represents the heat map of the proposed methods, showing clear distinction in quality. A significant lack of structures can be seen in panels a and b. SGBM parameters were varied without significantly improving RMSE (Fig. 8).

## Discussion

Fast, dense and accurate reconstructions of anatomical regions such as helix, antitragus, and antihelix are possible with Bayesian optimized NIR patterns. Adaptive NIR infrared patterns for microscopic surface reconstructions largely reduce homogeneities of the surgical site (skin, blood, bone, etc.) in the stereo image pairs captured by the stereo microscope [40]. Projection in near-infrared wavelengths allows to overcome some of the critical challenges in intraoperative stereo image reconstructions such as environmental lighting conditions and the intense surgical light sources used to illuminate the surgical site [41].

Further, the adaptive pattern addresses intraoperative usability by eliminating the need for moving the microscope, optimizing zoom due to lack of structures caused by the wrong pattern shape at the given zoom level, illumination caused by bad lightning at the given distance, repositioning the patient or changing reconstruction parameters manually to obtain accurate reconstructions of the surgical field.

The results confirm that, not surprisingly, strong environmental/surgical lighting in the operating room does significantly impact MiRe. In some regions, depth information was lost, while in other regions wrongly calculated, leading to lack of reconstruction details, in Fig. 7. The environmental light approach provided an inaccurate disparity map composed of false depth information, leading to inaccurate 3D point definition, as some parts were without useful textures, therefore homogeneous.

The entire adaptive pattern process was automatized without affecting the clinical workflow or the outcome of an intervention. The application with a calibrated microscope may be run fully automatically intraoperatively. Further optimization or adaptation to other stereo techniques using machine learning and synthetic data for disparity calculation might improve results [42]. This could improve assessment of disparity in regions which lack information. GPU CUDA [43] parallel processing could allow “real-time” quantitative evaluations and surgeon support.

Manual rotation of an object represents the weak point of the system due to the necessity to involve the user in the data collection process to assess the accuracy and perform calibration of the setup if needed. In the future, this could be resolved with a setup providing controlled linear and rotational motion of the object in the surgical scene. There are already approaches to perform the stereo rig calibration, but on the opposite camera side [44].

The adaptive pattern approach has shown its potential. However, the current setup of a standard surgical microscope does not allow changing its construction details (i.e., the stereo camera baseline). Our approach to project NIR patterns onto the surgical site can easily be further optimized by directly injecting the NIR patterns in the illumination pathway of a surgical microscope with minor technological efforts. This would lead to an optimal illumination of the surgical site at all zoom levels, reducing shadowing as the optical path is used directly. If necessary, microscope optics would benefit from NIR cutoff filter implementation in the oculars. Such improvements, however, are beyond the scope of this work.

The surgical microscope in use was a standard medical device that was extended by standard extraction cameras. It was calibrated for 5 zoom levels, which could keep the anatomical region (external ear) completely in the field of view. This is in general fine for predefined zooms levels in a laboratory setting; for intraoperative use, however, when the whole range of zoom levels is accessed or the microscope optics are changed by other means: simple camera realignment, beamsplitter reinsertion, etc., the current calibration might not prove adequate. For real surgical use, extended camera calibration would be necessary to allow for extrapolating a current zoom/focus setting from an extended lookup table. For research purposes, however, this would be beyond the scope of the current project. One might envision other approaches such as feature detection and pairwise correspondence matching which would allow calibration “on the fly” [45], and such features might even be projected from an adaptive pattern [46] or on screen visualized patterns [47, 48].

Extreme illumination conditions are regularly encountered in microscopic surgery including under- and over-illumination of the scene. This is well compensated by the human eye and modern video imagery, but can severely affect stereo reconstruction results due to the lack of depth information in such regions [39]. The adaptive pattern was projected from a micromirror-based DLP projector with a light source that does not interfere with OR environmental light, being visible by the two detection cameras of the microscope and the surgeon oculars in reduced intensity, with the improvement suggestions in the discussion. This device projected adaptive dot patterns, generated with a Bayesian optimizer based on RMSE between CT (ground truth) and MiRe closest points, to increase the amount of salient features and to decrease reconstruction inaccuracies by modification of the pattern parameters (dot size and dot distance), making it suitable for multiple zoom levels. The present results suggest that this approach can bring improvements in the reconstruction of demanding homogeneous anatomical regions while addressing different environmental scenarios without the necessity for surgeon input, therefore not impacting the default clinical workflow and providing a good base for quantification of a resected tissue.

The adaptive patterns presented here open up the possibility for further automatic evaluation of disparity maps to find regions with higher errors or missing depth information. This could be addressed, e.g., by locally refining the adaptive pattern to yield optimal stereo reconstructions of the surgical field. ArUco/X spot markers could be replaced by other multimodal ones or by software solutions as in [49].

Further clinical optimization close in cooperation with surgeons will provide a useful clinical tool at the end.

## Conclusion

The proposed method allows creation of stereo microscopic reconstructions of areas of relevance for microscopic surgeries at the lateral skull base. It overcomes the challenges of variable zoom levels, homogeneous surfaces and environmental illumination conditions via a customized NIR setup and a real-time intraoperative Bayesian optimized pattern exploiting the reconstruction error between co-registered ground truth and microscopic stereo reconstructions. To this end, however, introduction of the NIR light parallel to the microscope illumination path is a prerequisite to combine the complete surgical view with the exploitable NIR illuminated views. This was, however, out of the scope of this project. In the future, this approach could allow new approaches to monitor the progress of a surgery quantitatively in preoperative imagery.
